# Chiral Metal Coating to Enhance Water Electrolysis

**DOI:** 10.1021/acs.energyfuels.4c04304

**Published:** 2024-12-16

**Authors:** Deb Kumar Bhowmick, Nir Yuran, Michael Fadeev, Shira Yochelis, Yossi Paltiel, Ron Naaman

**Affiliations:** ‡ Department of Chemical and Biological Physics, Weizmann Institute of Science, Rehovot 7610001, Israel; § Department of Applied Physics, Center for Nanoscience and Nanotechnology, Hebrew University of Jerusalem, Jerusalem 91904, Israel; ∥ Chiral, Limited, Amal Street 12, Rosh Haayin 4809245, Israel

## Abstract

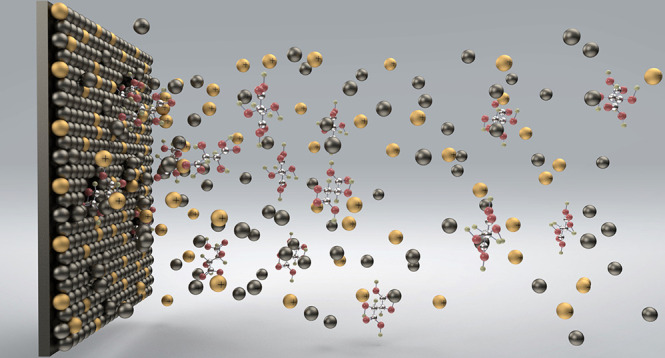

Producing hydrogen through water splitting often faces challenges of overpotential, stability, and expensive catalysts, which limit its efficiency and hinder the advancement of hydrogen production technologies. Nickel foam and nickel meshes have emerged as promising materials for electrolyzer electrodes due to their high surface area and the ability to produce electrolyzers with a very small gap between the anode and cathode. This study presents a simple method for coating Ni-based electrodes with a chiral Ni–Au film, using electroplating, thus enhancing its efficiency dramatically. We introduce chirality to the electroplating layer by incorporating an enantiopure chiral reagent into the electroplating solution. The chiral layer enhances the oxygen evolution reaction due to the chiral-induced spin selectivity effect. By optimizing the chiral electroplating process, we demonstrate the reduction of the overpotential and an increase in the reaction efficiency by 95% at 1 M KOH at room temperature.

Hydrogen is a clean and renewable energy source that has garnered significant attention recently due to its high gravimetric energy density (≈142 MJ kg^–1^), eco-friendly nature, and potential to reduce our reliance on fossil fuels and mitigate climate change.^1−4^ One of the most promising methods for hydrogen production is by water splitting, a process in which water is electrolyzed to produce hydrogen and oxygen.^5,6^ However, the efficiency of the water-splitting process is limited by overpotentials, particularly at the anode; this increases the energy required for the reaction and reduces the overall efficiency of hydrogen production.

At present, Ir/Ru oxides and Pt are considered the primary materials for electrocatalysis in the oxygen evolution reaction (OER) and the hydrogen evolution reaction (HER).^7^ However, the scarce abundance of these materials and their low chemical stability hinder reduction of the cost of hydrogen production.

Recent advancements in materials science have led to the development of foam metals as electrodes in electrolyzers.^8^ Foam metals, such as nickel foam, provide a high surface area, which increases the active sites for the electrochemical reaction and enables the use of zero-gap electrolyzers, further enhancing the process’s efficiency. Despite these improvements, the issue of overpotential remains a significant challenge.

It has been shown that overpotential occurs because the OER is a spin-forbidden process. Whereas water has a singlet ground state, the product, oxygen, has a triplet ground state.^9^ Hence, formally, this reaction is “forbidden”; namely, it has a spin-related potential barrier. Recent studies demonstrated a link between the formation of the oxygen triplet state, the overpotential, and the spin alignment.^4,10−12^ In the OER, two pairs of electrons are transferred.^13,14^ If the electrons in each pair have the same spin state, then the reaction is enhanced and the spin-related barrier is reduced. One method to obtain this spin co-alignment is by coating the anode with chiral material that serves as a spin filter due to the chiral-induced spin selectivity (CISS) effect. The CISS effect refers to spin-selective electron transport through chiral systems.^15−17^

In recent years, several methods for preparing chiral metal films have been reported.^4,18−21^ Among others, films of chiral gold and Ni have been produced. In the present study, electroplating is applied as a simple method for coating nickel foam and flat Ni–substrate electrodes with a chiral Ni–Au coating. We induce chirality in the electrodeposited composite metal layers by adding enantiopure chiral acids to the electroplating solution. The chiral layer formed on the anode induces the CISS effect, leading to a higher efficiency in the OER. We optimized the chiral electroplating process and investigated the impact of the chiral metal coating on the overpotential and efficiency of the OER with various organic chiral acids. Our findings provide new insights into designing more efficient and cost-effective electrodes for hydrogen production by electrolysis.

Several substrates were probed. In the first case, Ni–Au layers were electrochemically deposited using a solution of various salt mixtures, specifically Na_3_[Au(S_2_O_3_)_2_]·H_2_O, NiSO_4_, NiCl_2_, Na_2_S_2_O_3_, Na_2_SO_3_, and chiral/racemic tartaric acid at pH 6.5. The layer was grown chronoamperometrically on the flat Ni surface at a constant voltage of −0.9 V using Pt as the counter electrode and Hg/HgCl [saturated calomel electrode (SCE)] as the reference electrode.

The morphologies of the Ni–Au layers were measured by scanning electron microscopy (SEM). The low-magnification SEM image of chiral Ni–Au on a flat Ni surface (Figure 1a) shows the formation of a compact electrodeposited layer. The high-resolution SEM image (Figure 1b) shows amorphous structures with a large surface area. Figure 1c represents the SEM image of Ni–Au coated on another substrate, nickel foam. The image clearly shows the highly porous structure with a larger surface area than the flat surface. Furthermore, an energy-dispersive X-ray (EDX) presents a substantial abundance of Ni, Au, and C signals in the chiral Ni–Au-deposited layers (Figure 1d). Elemental mapping confirms the uniform distribution of the Ni, Au, and C elements on the entire surface (panels e, f, and g of Figure 1). The thickness of the deposited layer varies between 0.5 and 1 μm (see Figure S5 of the Supporting Information), and no change in the chirality or on the activity could be detected. The achiral layers have the same properties as the chiral layer.

**Figure 1:**
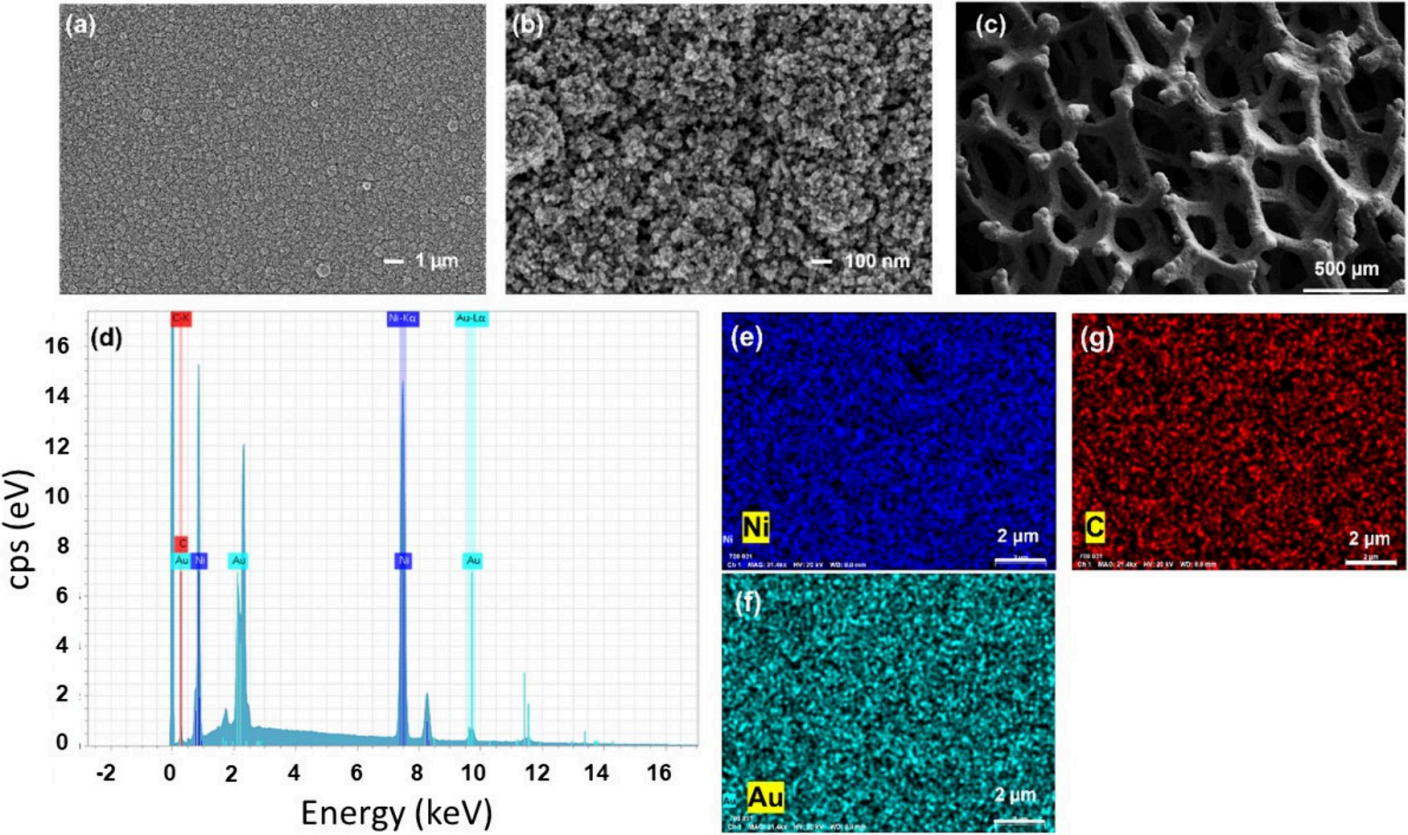
SEM images of a chiral Ni–Au layer on a flat Ni layer of a (a) surface area of 20 × 20 μm^2^ and (b) surface area of 2 × 2 μm^2^, (c) SEM image of chiral Ni–Au-coated Ni foam of a surface area of 2.5 × 2.5 mm^2^, (d) EDX spectrum of the chiral Ni–Au layer on a flat Ni layer, and (e, f, and g) EDX mapping of the Ni, Au, and C elements of the chiral Ni–Au layer on a flat Ni layer, respectively.

X-ray photoemission spectroscopy (XPS) was carried out to determine the surface chemical state of the Ni–Au layers. Figure 2 shows the high-resolution XPS spectra of Ni 2p, Au 4f, C 1s, and O 1s. The Ni 2p peak (Figure 2a) consists of two spin–orbit doublets at binding energies (BEs) of 856.3 and 874.0 eV, with a spin energy separation of 17.7 eV, corresponding to Ni 3p_3/2_ and Ni 3p_1/2_, respectively. The binding energies indicate that these two peaks correspond to a combination of the Ni^2+^ and Ni^3+^ states. Ni 2p and Ni^2+^ peaks appear at 857.3 and 875 eV, and the peaks corresponding to Ni^3+^ appear at 856.1 and 873.6 eV.^22^ Small peaks at binding energies of 853 and 870 eV are observed in the Ni^0^ state related to the nickel substrate. The peaks at BEs of 861.7 and 879.6 eV correspond to the shakeup peaks of Ni 2p_3/2_ and Ni 2p_1/2_. Ni^3+^ arises from Ni(OH)_2_ oxidation upon exposure to air. Figure 2b presents a high-resolution XPS spectrum of Au 4f with two spin doublets at 83.8 and 87.4 eV corresponding to 4f_7/2_ and 4f_5/2_. The presence of a Au 4f signal confirms the deposition of gold along with Ni. The atomic ratio of Ni and Au in the deposited layer is around 80:1 near the surface. Figure 2c presents the XPS profile of the osciometry spectrum of O 1s, where the peak is centered at 531.7 eV. The deconvoluted peak indicates that it is composed of three components. The peak at 529.5 eV corresponds to the band of oxygen in metal oxides (M–O), and the peak at 531.6 eV corresponds to hydroxides (M–OH). Here, it is Ni(OH)_2_, and the peak at 332.7 eV corresponds to oxygen in the carboxylic acid group of tartaric acid. Figure 2d represents the C 1s high-resolution XPS spectrum, which gives rise to four deconvoluted peaks corresponding to C–C at 284.9 eV from adventitious C, C–OH at 286.1 eV, O–C=O^–^ from carboxylic acid of tartaric acid at 287.8 eV, and COOH carboxylic acid of tartaric acid at 288.8 eV, as shown in refs (23 and 24). The study confirms that the electrodeposited layer consists of Ni and Au, where Ni is present in various oxidation states. Also, tartaric acid is present within the electrodeposited chiral metal layer.

**Figure 2:**
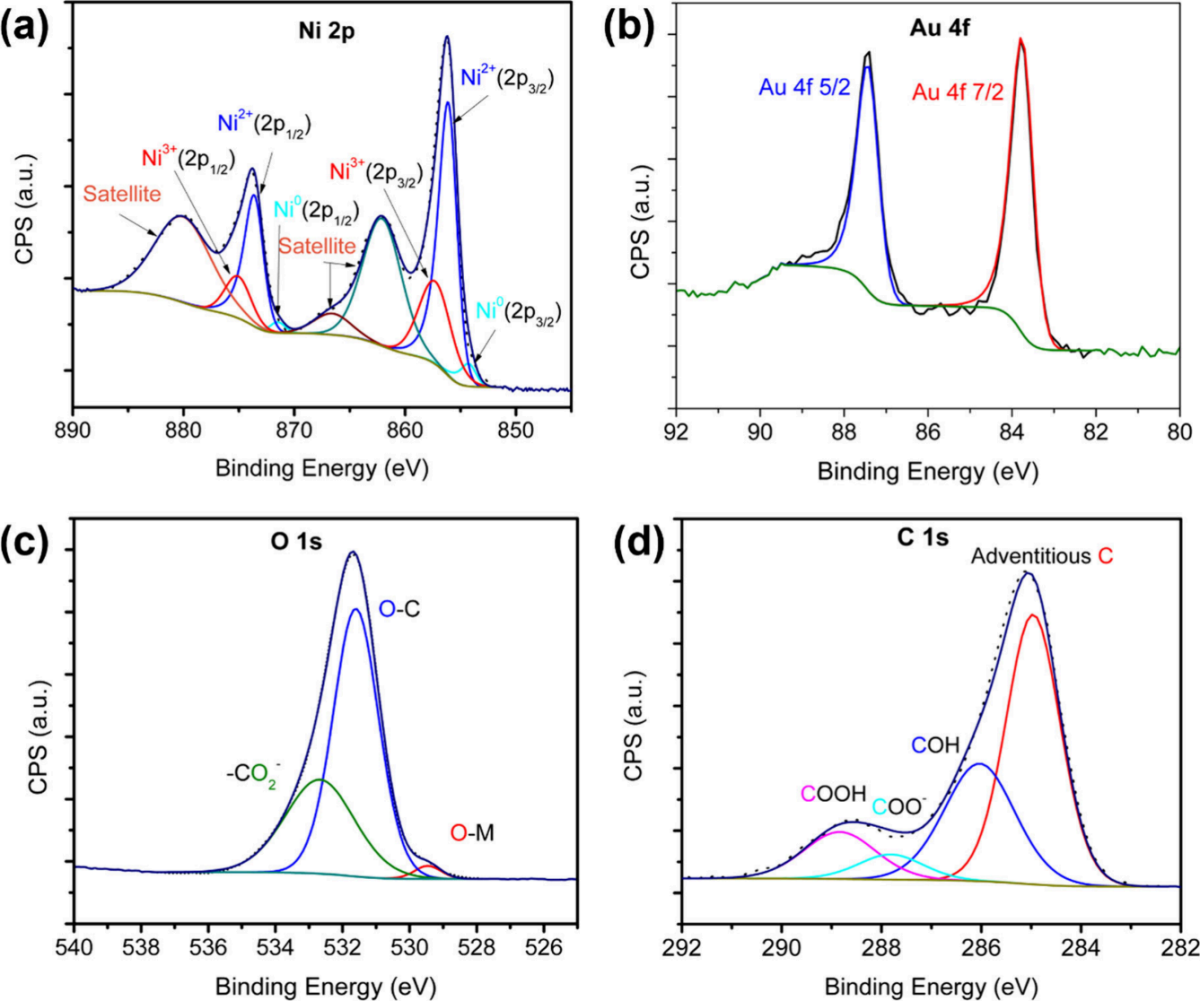
Deconvoluted high-resolution XPS spectra of (a) Ni 2p, (b) Au 4f, (c) O 1s, and (d) C 1s.

Solid-state circular dichroism (CD) measurements were performed on the Ni–Au layer to confirm the chirality of the electrodeposited layers. For this purpose, a thin Ni–Au composite layer was grown on an indium tin oxide (ITO) surface so that the surface remains semi-transparent. Figure 3 presents the CD spectra of different systems. The chiral Ni–Au layer, grown in the presence of either l- or d-tartaric acid, clearly shows CD signals ranging from 330 to 800 nm that are mirror images of each other. The racemic mixture shows no CD signal and bisects the CD signals that arise from the chiral layers. These results confirm that enantiopure tartaric acid induces chirality in the Ni–Au composite layer during electrodeposition.

**Figure 3:**
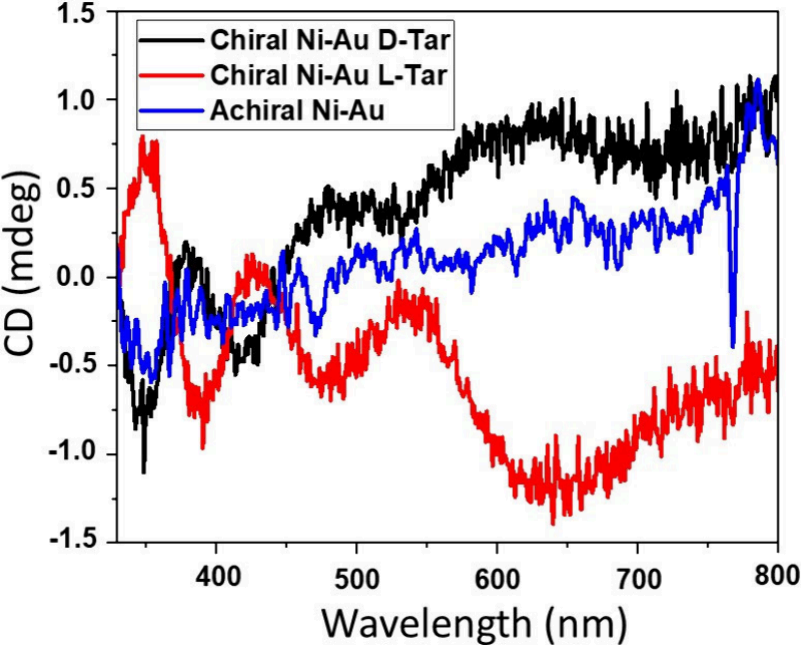
CD spectra of the chiral, achiral, and racemic Ni–Au layers in the solid state as a thin film on an ITO surface. The CD spectra are used for qualitative probing of the surface chirality.

All electrochemical results are presented with respect to a reversible hydrogen electrode (RHE). An electrode of 1 × 1 cm was used for electrochemical studies. The electrocatalytic performances of the Ni–Au layer-modified electrodes were studied in a 1.0 M KOH solution using cyclic voltammetry (CV).

## Electrochemical Studies on Flat Surfaces

**Figure 4:**
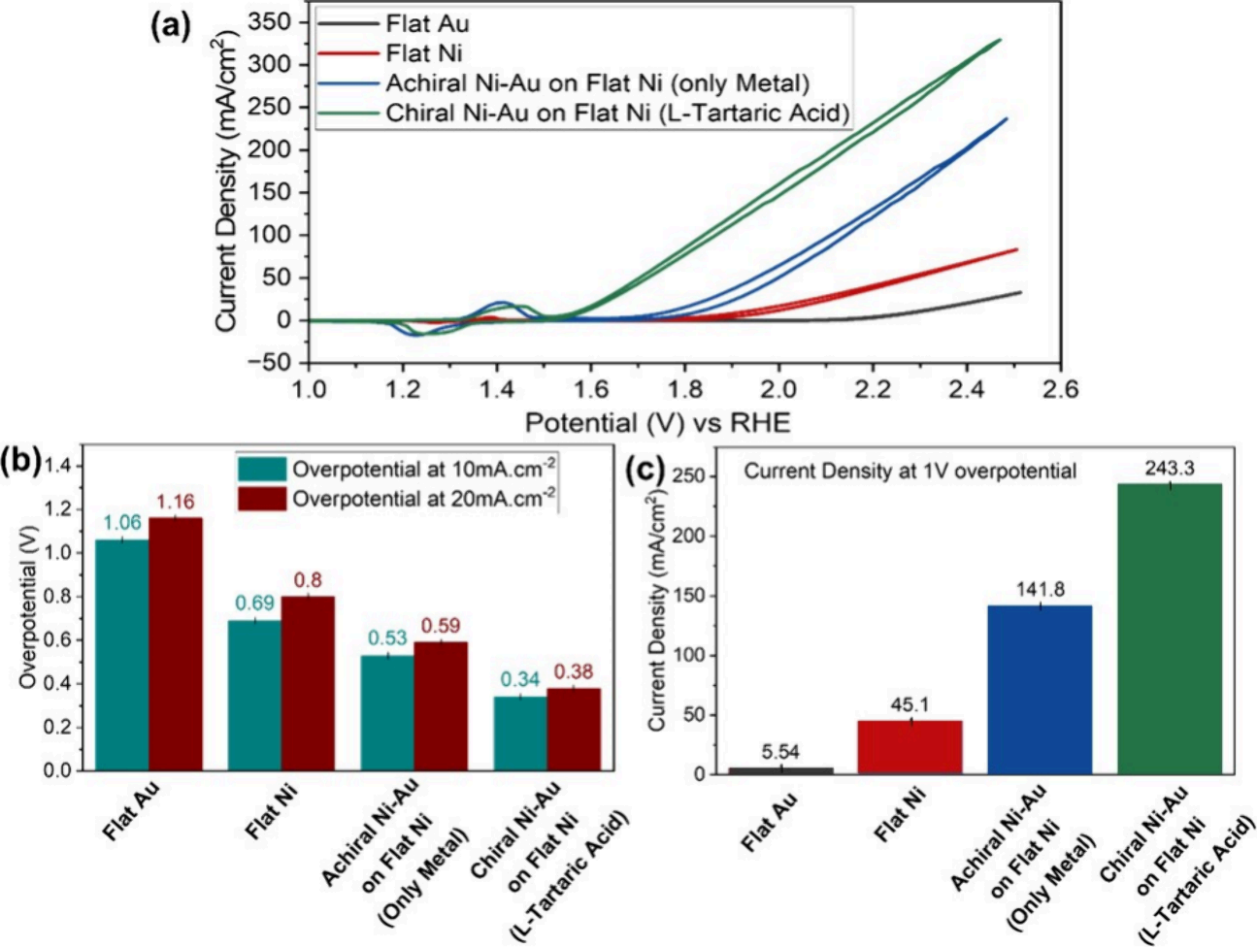
Electrochemical measurements on flat Si(100) with coating: (a) CV, (b) overpotential at 10 and 20 mA cm^–2^ current density, with the overpotential defined as the potential above 1.23 V versus RHE, and (c) current density at 2.23 V versus RHE (overpotential at 1.0 V) for an electrode coated with only Au, only Ni, achiral Ni–Au (only metal), and chiral Ni–Au (with l-tartaric acid).

All of the electrochemical studies were performed at least on four different samples with the same coating, and the error bars represent the deviations in their performance. Figure 4a presents the CV spectra obtained with various films grown on the Si(100) surface. It shows an apparent current enhancement and an overpotential reduction as the film changes from Au to Ni to achiral Ni–Au, and to the chiral Ni–Au surface. Along with the enhancement, the CV spectra show nickel oxidation and reduction peaks at 1.4 and 1.25 V versus RHE due to the transformation of Ni^2+^ → Ni^3+^ and Ni^3+^ → Ni^2+^, respectively. The conversion of Ni^2+^ to Ni^3+^ preactivates the composite layer, consequently catalyzing water splitting. The higher the surface density of the Ni^3+^ species, the higher the current associated with water splitting. Following preactivation, the flat Ni surface requires an overpotential of 0.69 and 0.8 V to obtain a current density of 10 and 20 mA/cm^2^, respectively (Figure 4b). The overpotential is defined as the potential above 1.23 V versus RHE. In contrast, the achiral Ni–Au layer requires an overpotential of only 0.53 and 0.59 V, and the chiral Ni–Au layer requires 0.34 and 0.38 V, respectively (Figure 4b). The overpotential is reduced by about 25% from Ni to the achiral Ni–Au surface and is reduced further by 35% from achiral Ni–Au to chiral Ni–Au. However, as shown in Figure 4c, the most significant current enhancement is observed in achiral Ni–Au and chiral Ni–Au, with around 200 and 400% improvement, respectively, compared to flat Ni surfaces. These results confirm that introducing Au into the grown Ni oxide layer improves the catalytic efficiency of Ni^3+^ for the water-splitting reaction. Introducing chirality to the system improves the OER efficiency even more, which is attributed to the CISS effect.

## Electrochemical Studies on Ni Foam

**Figure 5:**
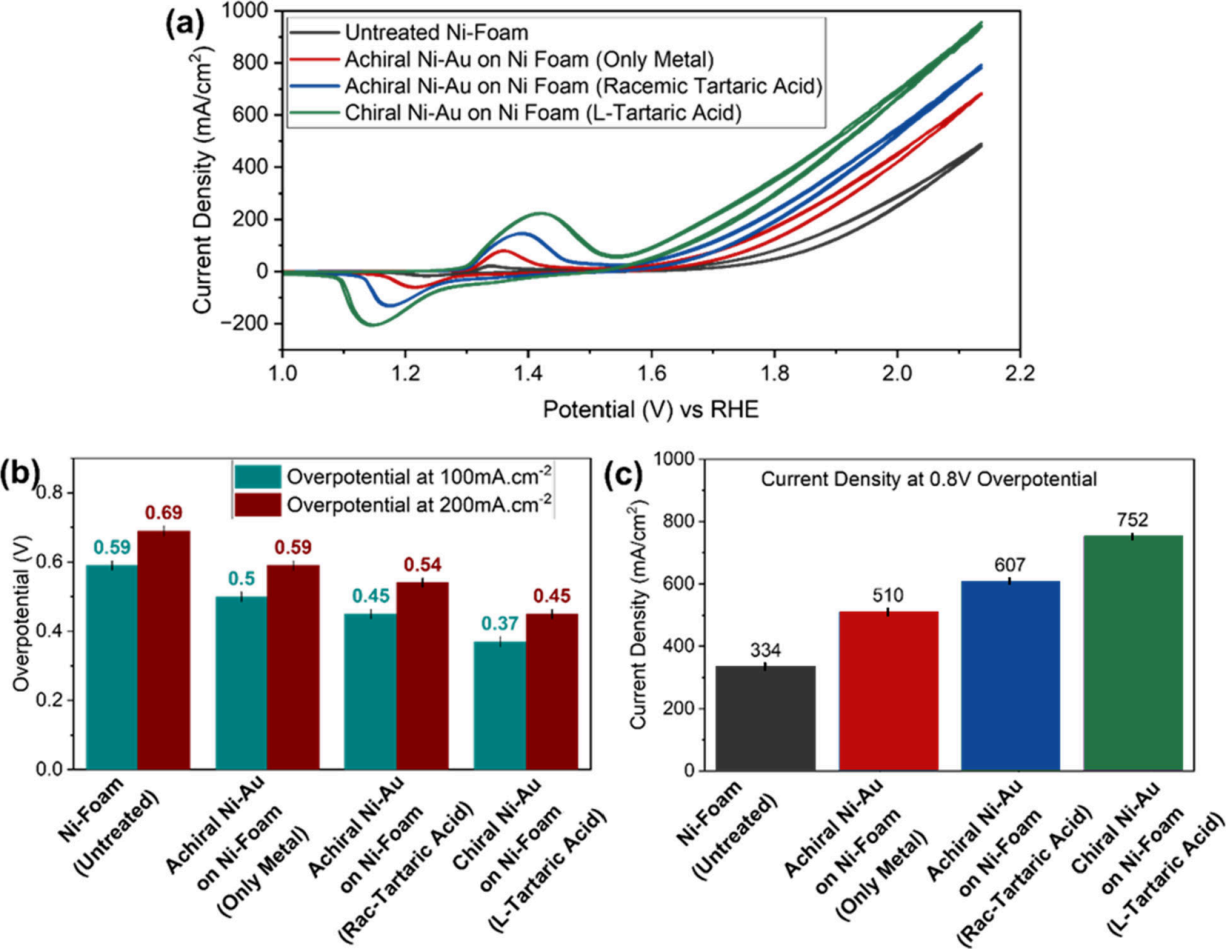
Electrochemical measurements on Ni foam: (a) CV, (b) overpotential at current densities of 100 and 200 mA cm^–2^, and (c) current density at a potential of 2.03 V versus RHE (overpotential of 0.8 V) of a bare Ni foam electrode and an electrode coated with achiral Ni–Au (only metal), achiral Ni–Au (racemic tartaric acid), and chiral Ni–Au (l-tartaric acid).

Figure 5a presents the CV measurements for chiral metal, racemic metal, and metal only, corresponding to samples with l-tartaric acid, l- + d-tartaric acid, and no chiral molecules, respectively. The study of the racemic mixture was introduced to determine whether the effect of the organic molecules results only from an increase in the surface area. In general, an increase in current was observed in the Ni–Au-coated nickel foam electrodes compared to the flat metals. This may result from increasing the surface area. Panels b and c of Figure 5 present the overpotentials and current densities, respectively. Clearly, the electrode coated with chiral Ni–Au provides the best results, with 95 and 40% improvement in current density, compared to the untreated and only metal samples, respectively. A similar trend was observed in the overpotentials, where the chiral Ni–Au electrode provided the lower overpotential.

Electrochemically active surface area (EASA) measurements were conducted for Ni foam, achiral Ni–Au (metal only), achiral Ni–Au (racemic tartaric acid), and chiral Ni–Au (l-tartaric acid) coatings (Figure S2 of the Supporting Information). The achiral Ni–Au surface showed a 10% increase in surface area compared to the chiral surface. This supports the conclusion that the primary effect, on the efficiency of the OER, arises from the CISS effect and its catalytic properties, rather than from changes in the electrode’s surface area.

**Figure 6:**
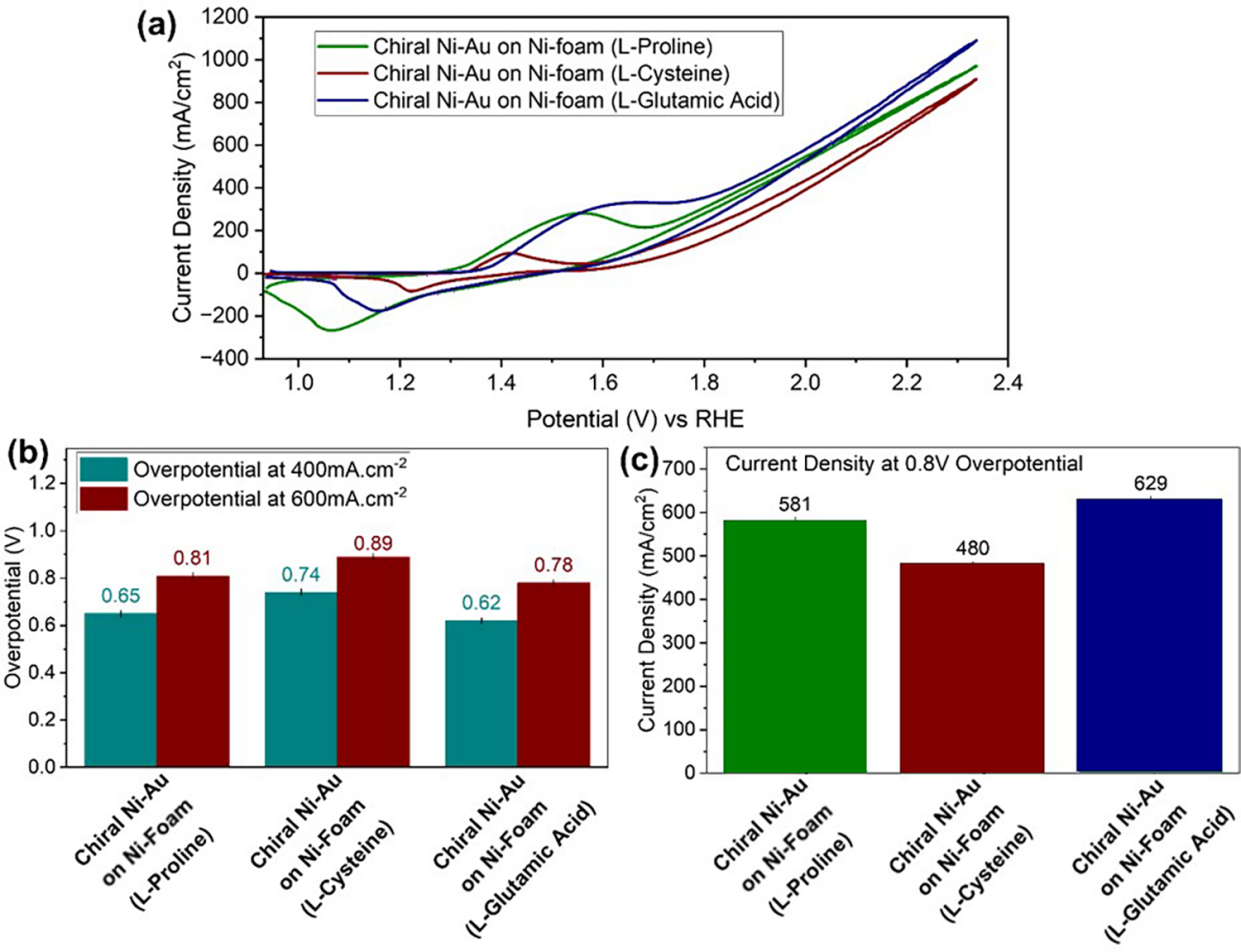
Electrochemical measurements of metal (Ni Foam) coated with proline, cysteine, and glutamic acid on Ni foam: (a) CV measurements of the coated electrodes, (b) calculated overpotential at 400 and 600 mA cm^–2^, and (c) current density at 2.03 V versus RHE.

To verify the importance of the structure of the chiral organic molecule on the activity of the chiral Ni–Au film, several amino acids were used in the electrodeposition process at the same concentration as tartaric acid. The molecules studied were proline, cysteine, and glutamic acid. Figure 6a presents the CV plots for those molecules. Figure 6b shows the overpotentials obtained for these three chiral Ni–Au layers at two different current densities of 400 and 600 mA cm^–2^.

All amino-acid-modified chiral layers show a reduction of overpotential compared to bare Ni foam, whereas glutamic acid shows the lowest overpotential. Figure 6c shows the current improvement at 2.03 V versus RHE based on the CV measurements. All of the Au–Ni layers with the amino acids show an improvement in current density compared to the values obtained before treatment, and the chiral Ni–Au sample showed a 60% increase in current density with glutamic acid compared to the result with the bare electrode. However, the results with tartaric acid surpass those obtained with glutamic acid.

**Figure 7:**
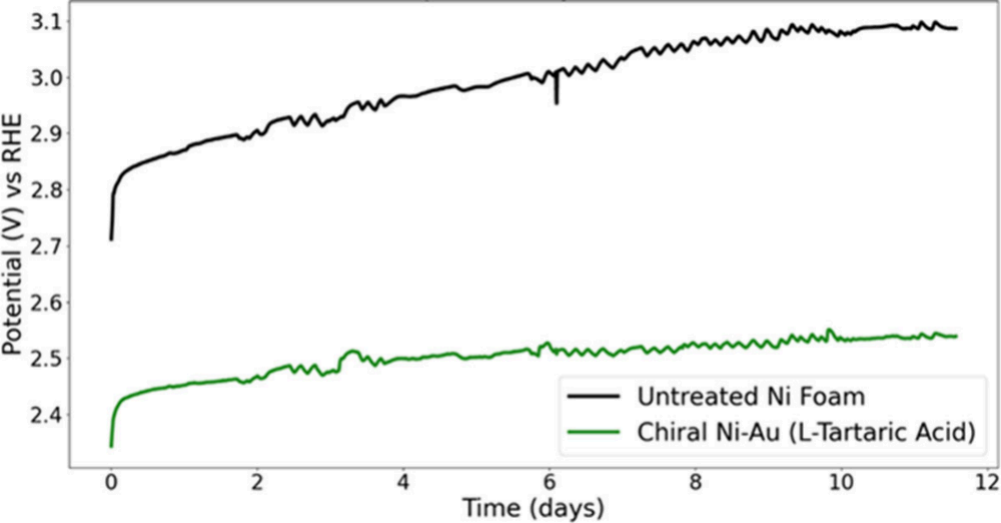
Chronopotentiometry measurement of the current during the OER. The current was set to 250 mA/cm^2^ in 5 M KOH solution. The anode was either untreated Ni foam (black) or Ni foam coated with chiral Ni–Au (l-tartaric acid) film.

The stability of the chiral metal coating was probed for 11.5 days and compared to that of the bare electrode (Figure 7). A chronopotentiometry measurement was conducted on untreated Ni foam and chiral Ni–Au (l-tartaric acid). Chiral Ni–Au stabilized after 1 day at 2.45 V versus RHE and, after 11 days, settled around 2.53 V versus RHE, reflecting an 80 mV increase over 10 days. In contrast, the untreated Ni foam reached 2.87 V versus RHE after 1 day and rose to 3.08 V after 11 days, showing a 210 mV increase over the same period. These findings indicate that the chiral Ni–Au coating provides protection to the Ni foam substrate in addition to enhancing its performance. It clearly shows that, beyond reducing the potential, the chiral coating improves the time stability of the electrode, and for the length of this study, there is no significant deterioration of the electrode’s performance.

The improved performance of the chiral Ni–Au coatings can be attributed to the CISS effect. The CISS effect occurs when chiral molecules or structures preferentially transmit electrons with one spin state. In the context of water electrolysis, the chiral layer on the anode enables the transfer of electrons with one specific spin state from OH^–^ in the solution, leaving the OH radicals with unpaired electrons in the same spin state. Therefore, when two radicals interact to form oxygen, they do so on a triplet spin state potential, which allows the formation of oxygen in its triplet ground state.^10^ This selective interaction reduces the overpotential required for the OER by facilitating more efficient electron transfer.

This study demonstrates the significant potential of chiral metal coatings in enhancing the efficiency of water electrolysis for hydrogen production. By employing a straightforward electroplating method, we successfully introduced chirality into Ni–Au coatings on nickel foam and flat nickel electrodes; this chirality introduces the CISS effect. The enhancement in the OER is expressed in a substantial reduction in the overpotential and a notable increase in the OER efficiency. Our findings highlight the feasibility of using chiral metal coatings to address the challenges of overpotential and efficiency in water-splitting technologies, paving the way for more cost-effective and sustainable hydrogen production methods.

## Supporting Information Available

Description of the layer deposition and the electrochemical measurements procedure, description of the characterization methods, and data obtained from the characterization as well as additional control experiments (PDF)

## References

[ref1] Dresselhaus M. S.; Thomas I. L. Alternative Energy Technologies. Nature 2001, 414, 332–337. 10.1038/35104599. 11713539

[ref2] Seh Z. W.; Kibsgaard J.; Dickens C. F.; Chorkendorff I.; Nørskov J. K.; Jaramillo T. F. Combining Theory and Experiment in Electrocatalysis: Insights into Materials Design. Science 2017, 355, eaad4998 10.1126/science.aad4998. 28082532

[ref3] Schlapbach L.; Züttel A. Hydrogen-Storage Materials for Mobile Applications. Nature 2001, 414, 353–358. 10.1038/35104634. 11713542

[ref4] Vadakkayil A.; Clever C.; Kunzler K. N.; Tan S.; Bloom B. P.; Waldeck D. H. Chiral Electrocatalysts Eclipse Water Splitting Metrics through Spin Control. Nat. Commun. 2023, 14, 1067 10.1038/s41467-023-36703-w. 36828840 PMC9958132

[ref5] Tang T.; Jiang W. J.; Niu S.; Liu N.; Luo H.; Chen Y. Y.; Jin S. F.; Gao F.; Wan L. J.; Hu J. S. Electronic and Morphological Dual Modulation of Cobalt Carbonate Hydroxides by Mn Doping toward Highly Efficient and Stable Bifunctional Electrocatalysts for Overall Water Splitting. J. Am. Chem. Soc. 2017, 139, 8320–8328. 10.1021/jacs.7b03507. 28535047

[ref6] Yang L.; Yu G.; Ai X.; Yan W.; Duan H.; Chen W.; Li X.; Wang T.; Zhang C.; Huang X.; Chen J.-S.; Zou X. Efficient Oxygen Evolution Electrocatalysis in Acid by a Perovskite with Face-Sharing IrO_6_ Octahedral Dimers. Nat. Commun. 2018, 9, 5236 10.1038/s41467-018-07678-w. 30531797 PMC6286314

[ref7] Reier T.; Oezaslan M.; Strasser P. Electrocatalytic Oxygen Evolution Reaction (OER) on Ru, Ir, and Pt Catalysts: A Comparative Study of Nanoparticles and Bulk Materials. ACS Catal. 2012, 2, 1765–1772. 10.1021/cs3003098.

[ref8] Bidault F.; Brett D. J. L.; Middleton P. H.; Abson N.; Brandon N. P. A New Application for Nickel Foam in Alkaline Fuel Cells. Int. J. Hydrogen Energy 2009, 34, 6799–6808. 10.1016/j.ijhydene.2009.06.035.

[ref9] Plante I. Energetic and Chemical Reactivity of Atomic and Molecular Oxygen, June 28, 2010; https://three.jsc.nasa.gov/articles/RadChemO2Sidebar.pdf.

[ref10] Zhang W.; Banerjee-Ghosh K.; Tassinari F.; Naaman R. Enhanced Electrochemical Water Splitting with Chiral Molecule-Coated Fe_3_O_4_ Nanoparticles. ACS Energy Lett. 2018, 3, 2308–2313. 10.1021/acsenergylett.8b01454.

[ref11] Mtangi W.; Kiran V.; Fontanesi C.; Naaman R. Role of the Electron Spin Polarization in Water Splitting. J. Phys. Chem. Lett. 2015, 6, 4916–4922. 10.1021/acs.jpclett.5b02419. 26615833 PMC4685426

[ref12] Mtangi W.; Tassinari F.; Vankayala K.; Vargas Jentzsch A.; Adelizzi B.; Palmans A. R. A.; Fontanesi C.; Meijer E. W.; Naaman R. Control of Electrons’ Spin Eliminates Hydrogen Peroxide Formation during Water Splitting. J. Am. Chem. Soc. 2017, 139, 2794–2798. 10.1021/jacs.6b12971. 28132505 PMC5330654

[ref13] Raveendran A.; Chandran M.; Dhanusuraman R. A Comprehensive Review on the Electrochemical Parameters and Recent Material Development of Electrochemical Water Splitting Electrocatalysts. RSC Advances. 2023, 13, 3843–3876. 10.1039/D2RA07642J. 36756592 PMC9890951

[ref14] Liang Y.; Banjac K.; Martin K.; Zigon N.; Lee S.; Vanthuyne N.; Garcés-Pineda F. A.; Galán-Mascarós J. R.; Hu X.; Avarvari N.; Lingenfelder M. Enhancement of electrocatalytic oxygen evolution by chiral molecular functionalization of hybrid 2D electrodes. Nat. Commun. 2022, 13, 3356 10.1038/s41467-022-31096-8. 35688831 PMC9187664

[ref15] Bloom B.; Paltiel Y.; Naaman R.; Waldeck D. Chiral Induced Spin Selectivity. Chem. Rev. 2024, 124, 1950–1991. 10.1021/acs.chemrev.3c00661. 38364021 PMC10906005

[ref16] Amsallem D.; Kumar A.; Naaman R.; Gidron O. Spin Polarization through Axially Chiral Linkers: Length Dependence and Correlation with the Dissymmetry Factor. Chirality 2023, 35, 562–568. 10.1002/chir.23556. 36896481

[ref17] Kettner M.; Göhler B.; Zacharias H.; Mishra D.; Kiran V.; Naaman R.; Fontanesi C.; Waldeck D. H.; Sek S.; Pawłowski J.; Juhaniewicz J. Spin Filtering in Electron Transport Through Chiral Oligopeptides. J. Phys. Chem. C 2015, 119, 14542–14547. 10.1021/jp509974z.

[ref18] Behar-Levy H.; Neumann O.; Naaman R.; Avnir D. Chirality Induction in Bulk Gold and Silver. Adv. Mater. 2007, 19, 1207–1211. 10.1002/adma.200601702.

[ref19] Ma W.; Xu L.; De Moura A. F.; Wu X.; Kuang H.; Xu C.; Kotov N. A. Chiral Inorganic Nanostructures. Chem. Rev. 2017, 117, 8041–8093. 10.1021/acs.chemrev.6b00755. 28426196

[ref20] Vensaus P.; Liang Y.; Zigon N.; Avarvari N.; Mujica V.; Soler-Illia G. J. A. A.; Lingenfelder M. Hybrid mesoporous electrodes evidence CISS effect on water oxidation. J. Chem. Phys. 2024, 160, 111103 10.1063/5.0199339. 38511663

[ref21] Bian Z.; Kato K.; Ogoshi T.; Cui Z.; Sa B.; Tsutsui Y.; Seki S.; Suda M. Hybrid Chiral MoS_2_ Layers for Spin-Polarized Charge Transport and Spin-Dependent Electrocatalytic Applications. Advanced Science 2022, 9, 2201063 10.1002/advs.202201063. 35481673 PMC9189682

[ref22] Xiong D.; Li W.; Liu L. Vertically Aligned Porous Nickel(II) Hydroxide Nanosheets Supported on Carbon Paper with Long-Term Oxygen Evolution Performance. Chem. - Asian J. 2017, 12, 543–551. 10.1002/asia.201601590. 28052617

[ref23] Arrigo R.; Gallarati S.; Schuster M. E.; Seymour J. M.; Gianolio D.; da Silva I.; Callison J.; Feng H.; Proctor J. E.; Ferrer P.; Venturini F.; Grinter D.; Held G. Influence of Synthesis Conditions on the Structure of Nickel Nanoparticles and Their Reactivity in Selective Asymmetric Hydrogenation. ChemCatChem 2020, 12 (5), 1491–1503. 10.1002/cctc.201901955.

[ref24] Baldanza S.; Ardini J.; Giglia A.; Held G. Stereochemistry and Thermal Stability of Tartaric Acid on the Intrinsically Chiral Cu{531} Surface. Surf. Sci. 2016, 643, 108–116. 10.1016/j.susc.2015.08.021.

