# Supplementary material for: Chiral Metal Coating to Enhance Water Electrolysis
Source: Energy Fuels. 2024 Dec 16;39(1):764–70. doi: 10.1021/acs.energyfuels.4c04304 (PMC11726431; doi:10.1021/acs.energyfuels.4c04304)
Supplement: Supplementary file 1 — ef4c04304_si_001.pdf [file ef4c04304_si_001.pdf]

Supplementary Information

Chiral metal coating to enhance water electrolysis

Deb Kumar Bhowmick[1#], Nir Yuran[2#], Michael Fadeev,[3] Shira Yochelis[2], Yossi Paltiel[2], and Ron Naaman[1*]

1) Department of Chemical and Biological Physics, Weizmann Institute of Science, Rehovot 7610001, Israel
2) Department of Applied Physics, Center for Nanoscience and Nanotechnology, Hebrew University of Jerusalem, Jerusalem 91904, Israel
3) Chiral Ltd, Amal St. 12, Rosh Hain 4809245 Israel.

Layer Depositions: Electrochemical depositions have been performed mainly with two types of substrates: flat substrates, where metal layers are grown on Si(100) surfaces, and another system, nickel foam. Four types of metal-coated layers were used for the OER studies on flat surfaces.

Si(100) wafers were cut into rectangular shapes of (1× 4) cm^2, and then 100 nm Gold or 120 nm Nickel were grown using an E-beam evaporator. Furthermore, Ni-coated surfaces were used for electroplating the Ni-Au composite materials. In that case, the Ni-coated surfaces were cleaned in boiling acetone and ethanol, each for 10 min before electrodeposition.

The Ni foam was cut into 1×1±0.1 cm^2 squares and then subjected to a thorough cleaning process prior to any pretesting and electroplating. The cleaning procedure involved washing the foam with pure ethanol, followed by a 2-minute exposure to O_2 plasma at 100W for each side, a 5-minute sonication in 37% (v/v) HCl, rinsing with distilled water to remove any residues, and a final 5-minute sonication in ethanol.

The electroplating was conducted using a three-electrode system, comprising the Ni-Foam as the working electrode, a Pt wire as the counter electrode, and an Ag/AgCl reference electrode. Cyclic voltammetry was performed, scanning from -1.4V to -0.6V at a rate of 50 mV/s. The electroplating solution contained $0.02\text{M}\ \text{Na}_3\text{Au}(\text{S}_2\text{O}_3)_2$, $0.25\text{M}\ \text{Ni}(\text{SO}_4)_2 \cdot 6\text{H}_2\text{O}$, $0.05\text{M}\ \text{NiCl}_2$, $0.42\text{M}\ \text{Na}_2\text{S}_2\text{O}_3$, $0.42\text{M}\ \text{Na}_2\text{SO}_3$, and a 0.6M chiral molecule in distilled water; the final pH of the solution was adjusted using NaOH resulting in 6.5 ± 0.1. A reference anode was made, using a racemic chiral in the same concentration, and without organic chiral material (metal only) to determine the effect of CISS in the process.

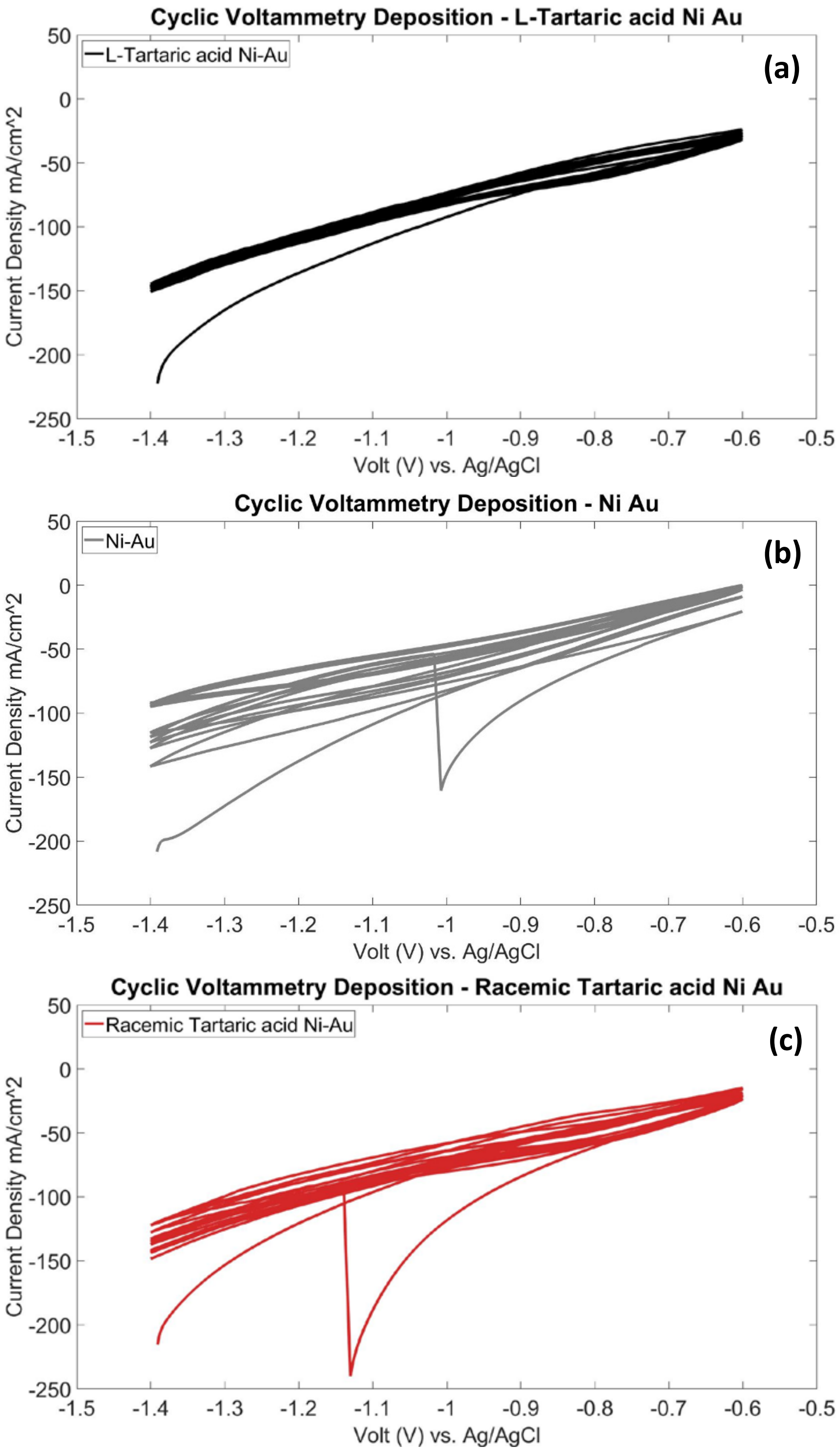

Figure S1: Electrodeposition of (a) Ni Au (Only Metal), (b) Racemic Tartaric acid Ni Au, and (c) L-Tartaric acid Ni Au (Chiral Metal).

Electrochemical Study: The electrochemical characterizations were conducted in two separate systems. Initially, cyclic voltammetry (CV) was performed in a three-electrode setup with a Ti net as the counter electrode for the Ni foam substrates, Pt as a counter electrode for flat substrates, and an Hg/HgO electrode (purchased from ALC Co.) as the reference electrode. A pretesting CV was carried out for all electrodes before the deposition process (and after cleaning) to minimize potential errors arising from variations in electrode size. Subsequently, the coated electrodes were tested for long-term constant voltage measurements. This setup tests the coating's stability under prolonged and harsh conditions. Both measurements were done in $1M\ KOH$.

All the experiments were performed at 24-25ºC under ambient conditions. In this work, all potentials reported were adjusted to the reversible hydrogen electrode (RHE). For RHE conversion at different pH, the following equation was used.

$$E(RHE) = E(Hg/HgO) + E^0(Hg/HgO) + 0.059pH$$

At pH 14,

$$E^0(Hg/HgO) + 0.059pH = 0.936$$

We defined the overpotential at a specific current density as the extra potential required to achieve that current density with respect to the minimum potential required by the thermodynamic potential, whose reversible thermodynamic potential is 1.23V vs RHE for OER.

Characterization:

X-ray photoelectron spectroscopy (XPS): XPS measurements were carried out using a KratosAxis Ultra DLD spectrometer equipped with a monochromatic Al Kα X-ray source (hv = 1486.6 eV) operating at 75 W. Measurements were performed at the surface normal. Elemental concentrations were measured from the relative intensities of different elements. XPS spectra were analyzed using CASA XPS software using a Shirley background correction. The spectra were fitted with the minimum number of peaks needed to reproduce the spectral features using Gaussian−Lorentzian product function.

Scanning electron microscopy (SEM): Morphological investigations were performed on a high-resolution scanning electron microscope (HRSEM, Carl Zeiss Ultra Plus). Energy-dispersive X-ray spectroscopy (EDS) was performed using a spectrometer (Oxford Instruments X-MaxN) attached to a SEM.

X-ray Diffraction (XRD): The powder X-ray diffraction pattern (PXRD) was recorded using a Bruker AXS with D8 advance with Cu Ka radiation (1.54 Å), and with a step size of 0.02 in a 2θ range of 5–800.

Circular Dichroism (CD) Spectroscopy: The Circular Dichroism spectroscopy measurements were performed using a Chirascan spectrometer with a thermoelectrically controlled single-cell holder. The measurements were performed at 1 s time per point, a 1 nm step size and with a bandwidth of 1 nm.

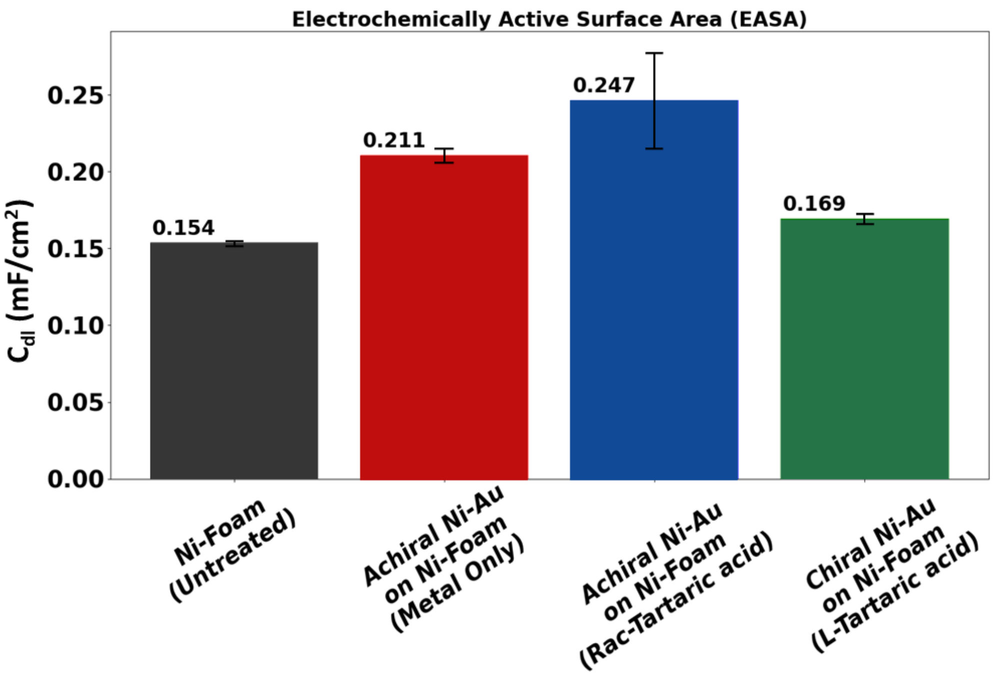

Figure S2: EASA measurements of untreated Ni-Foam, Achiral Ni-Au on Ni-Foam (Metal Only), Achiral Ni-Au on Ni-Foam (Rac-Tartaric acid), and Chiral Ni-Au on Ni-Foam (L-Tartaric acid). Average of 3 samples in each batch.

An EASA measurement done on Ni-Foam for understanding the changes in the active surface area of the Ni-Foam after treatment. The measurements done in a three-electrode setup in 0.1M KOH, Ni-Foam and Hg/HgO sat. as counter and reference electrodes, respectively. The cyclic voltammetry scan done $\pm 0.1V$ around the OCP. Rate scan are 10, 20, 30, 40, 50, 60, 70, 80, 90 mV/s.

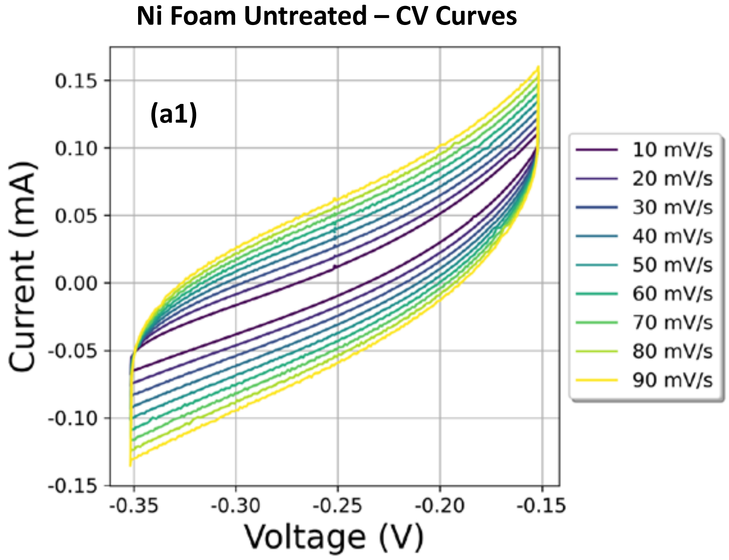
Ni Foam Untreated – CV Curves
(a1)
Current (mA)
Voltage (V)
0.15
0.10
0.05
0.00
-0.05
-0.10
-0.15
-0.35
-0.30
-0.25
-0.20
-0.15
10 mV/s
20 mV/s
30 mV/s
40 mV/s
50 mV/s
60 mV/s
70 mV/s
80 mV/s
90 mV/s

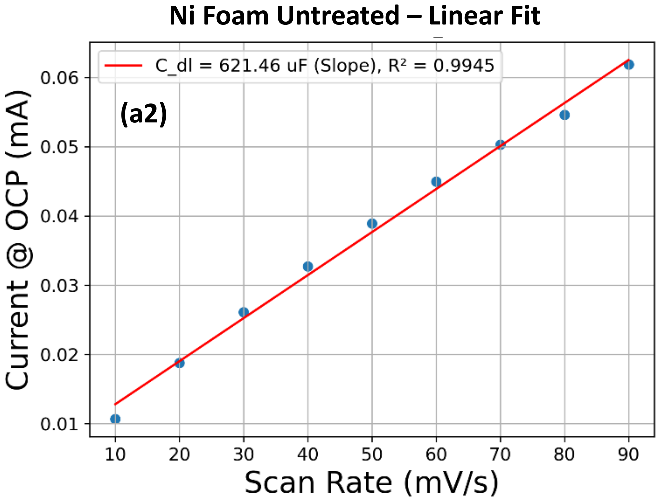
Ni Foam Untreated – Linear Fit
C_dl = 621.46 uF (Slope), R² = 0.9945
(a2)
Current @ OCP (mA)
Scan Rate (mV/s)
0.06
0.05
0.04
0.03
0.02
0.01
10
20
30
40
50
60
70
80
90

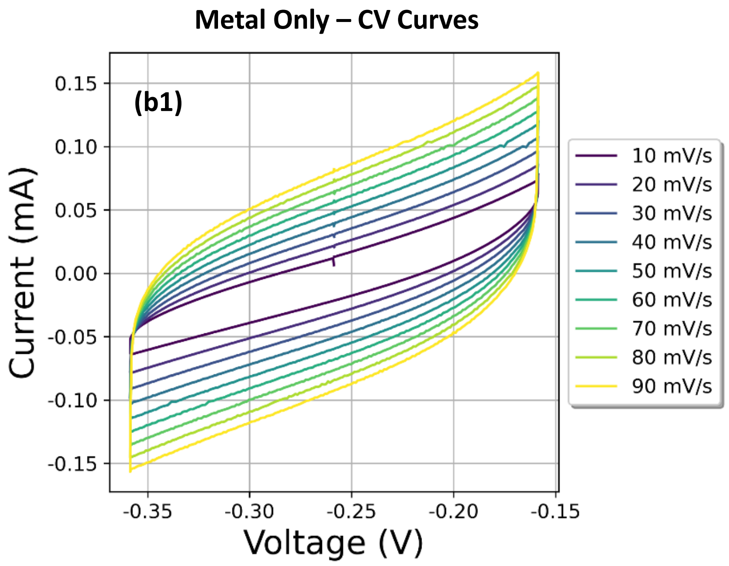
Metal Only – CV Curves
(b1)
Current (mA)
Voltage (V)
0.15
0.10
0.05
0.00
-0.05
-0.10
-0.15
-0.35
-0.30
-0.25
-0.20
-0.15
10 mV/s
20 mV/s
30 mV/s
40 mV/s
50 mV/s
60 mV/s
70 mV/s
80 mV/s
90 mV/s

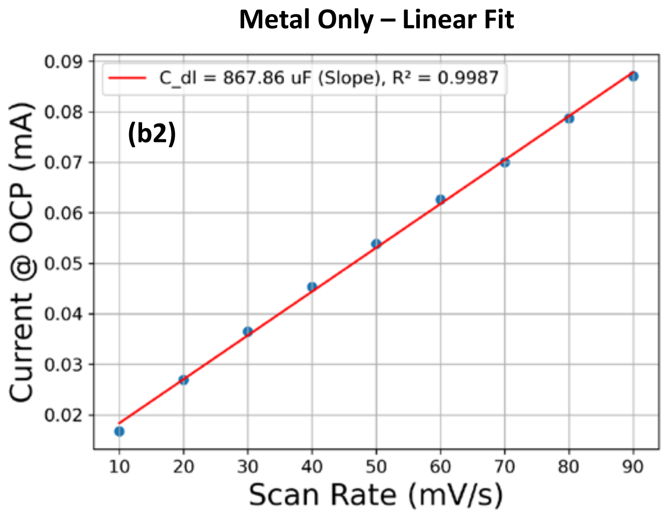
Metal Only – Linear Fit
C_dl = 867.86 uF (Slope), R² = 0.9987
(b2)
Current @ OCP (mA)
Scan Rate (mV/s)
0.09
0.08
0.07
0.06
0.05
0.04
0.03
0.02
10
20
30
40
50
60
70
80
90

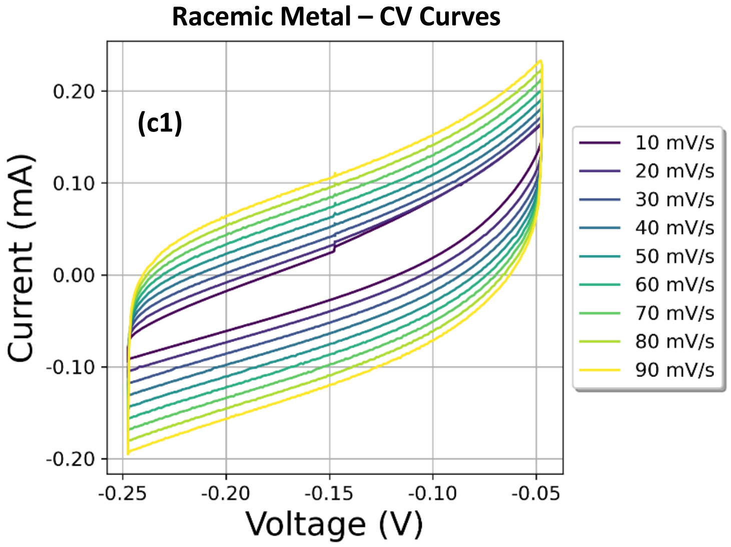
Racemic Metal – CV Curves
(c1)
Current (mA)
Voltage (V)
0.20
0.10
0.00
-0.10
-0.20
-0.25
-0.20
-0.15
-0.10
-0.05
10 mV/s
20 mV/s
30 mV/s
40 mV/s
50 mV/s
60 mV/s
70 mV/s
80 mV/s
90 mV/s

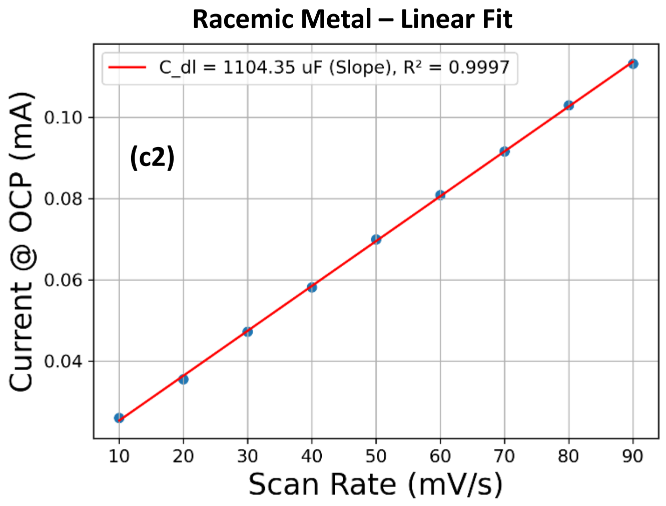
Racemic Metal – Linear Fit
C_dl = 1104.35 uF (Slope), R² = 0.9997
(c2)
Current @ OCP (mA)
Scan Rate (mV/s)
0.10
0.08
0.06
0.04
10
20
30
40
50
60
70
80
90

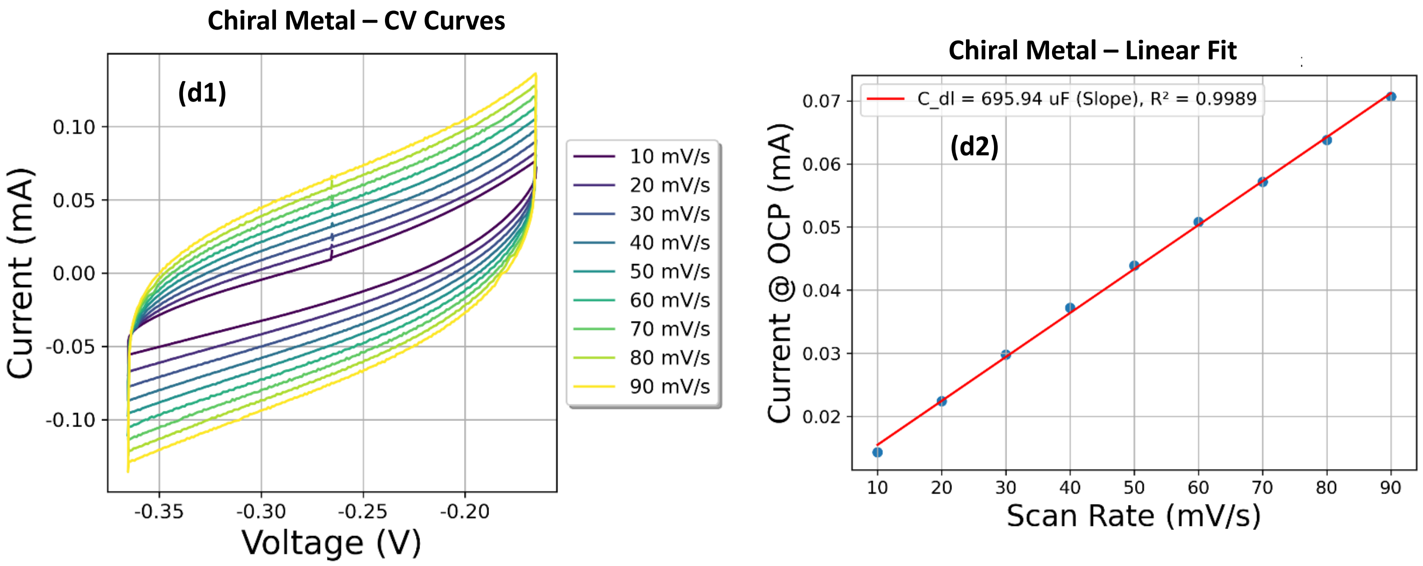

Figure S3. CV curves and linear fitting of (a1) & (a2) untreated Ni-Foam, (b1) & (b2) Achiral Ni-Au on Ni-Foam (Metal Only), (c1) & (c2) Achiral Ni-Au on Ni-Foam (Rac-Tartaric acid), and (d1) & (d2) Chiral Ni-Au on Ni-Foam (L-Tartaric acid). Average of 3 samples in each batch.

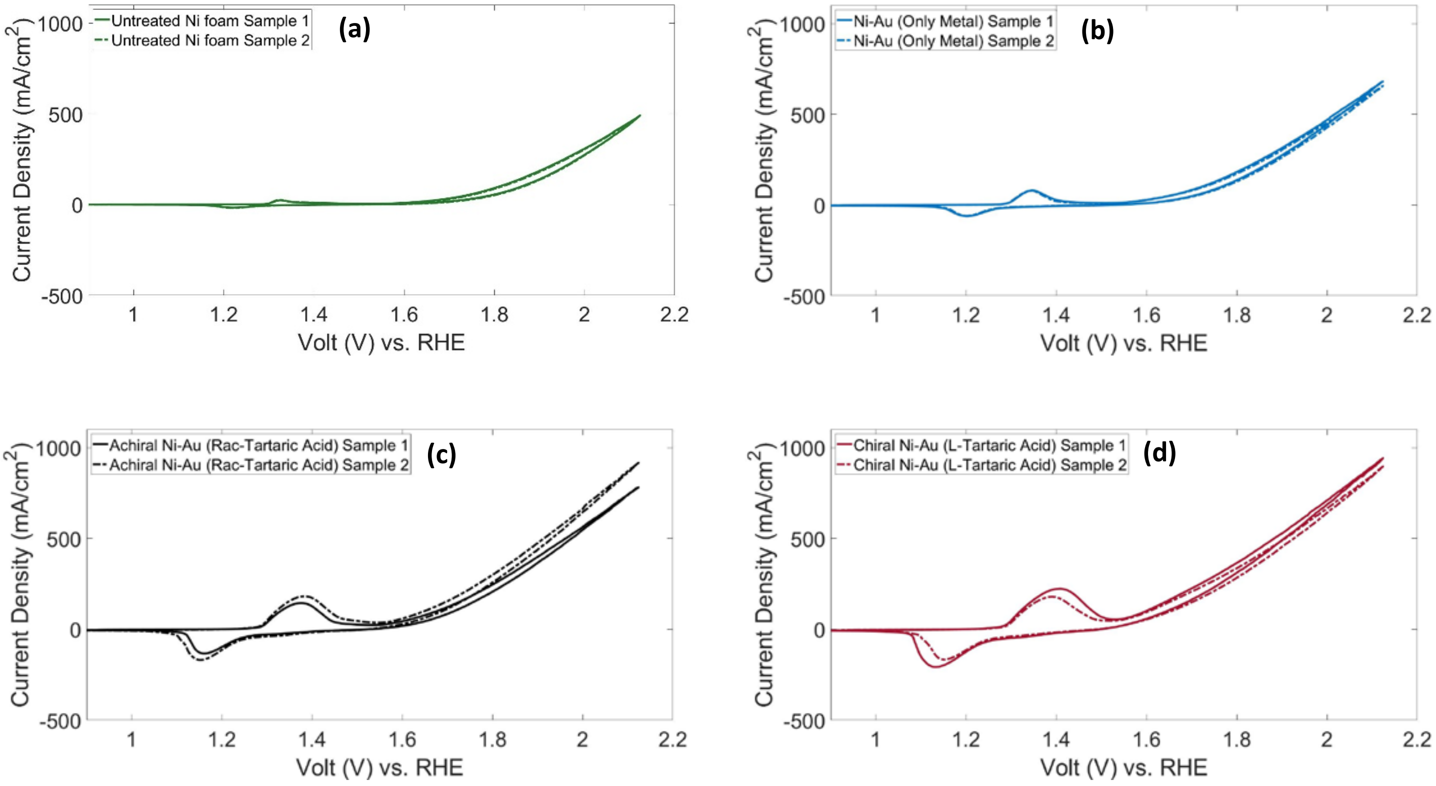

Figure S4. CV curves of 2 samples of (a) untreated Ni-Foam,(b) Achiral Ni-Au on Ni-Foam (Metal Only), (c) Achiral Ni-Au on Ni-Foam (Rac-Tartaric acid), and (d) Chiral Ni-Au on Ni-Foam (L-Tartaric acid).

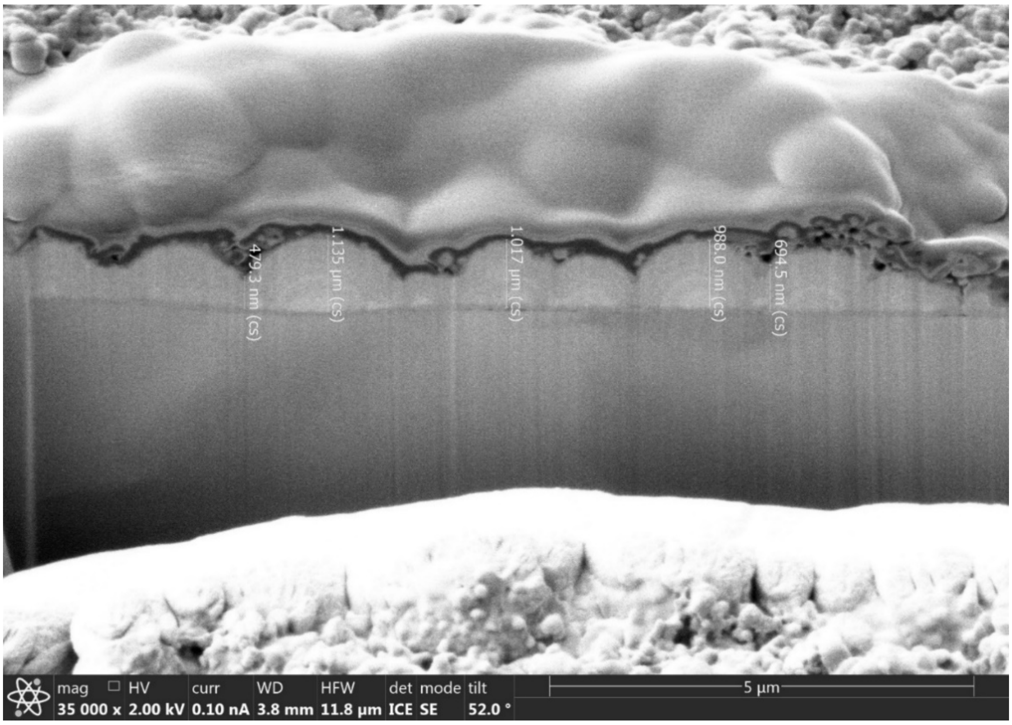

Figure S5. A) Scanning electron microscopy (SEM) cross section of chiral Ni-Au (L-Tartaric acid) done by focused ion beam (FIB). The Chiral Ni-Au average coating size is approximately $0.75\mu m$. Identical images were obtained for the Ni-Au without chiral molecules and with racemic mixture.

Figure S6 summarizes the various results obtained from the electrochemical measurements conducted on Ni-foam electrode, either bare or coated with achiral or chiral Ni/Au coating.

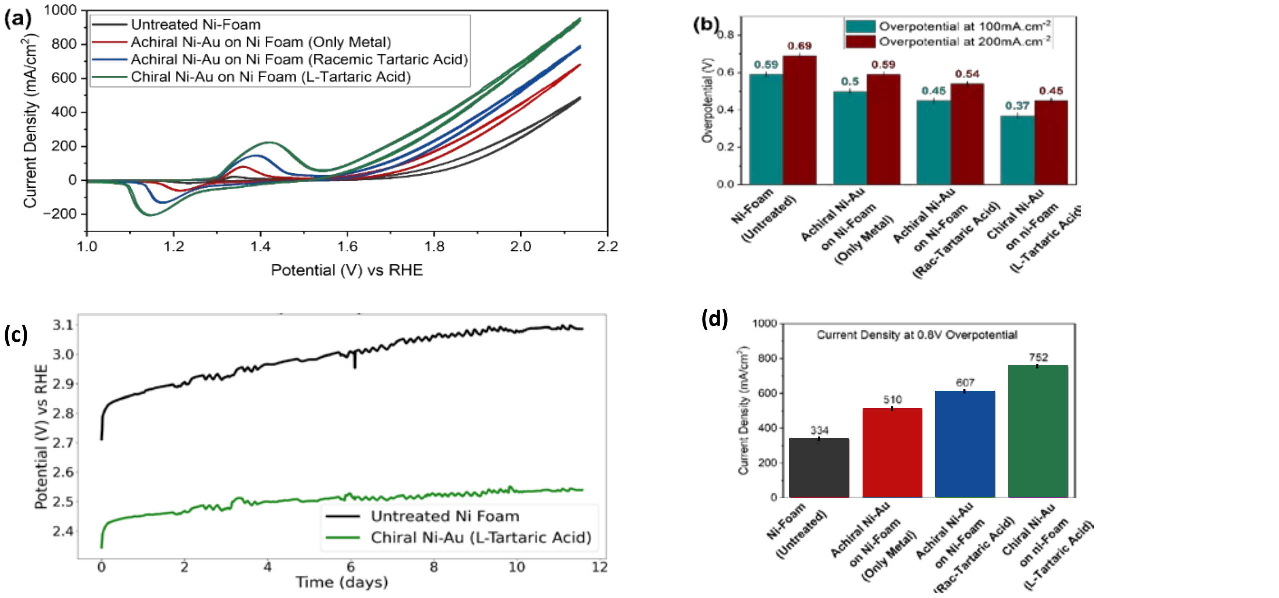

Figure S6: Electrochemical measurements on Ni-foam. (a) cyclic voltammetry, (b) overpotential at current densities of 100 and 200 mA·cm^2. (c) Chronopotentiometry measurement at 250mA/cm2 in 5M KOH of Untreated Ni foam, and Chiral Ni-Au (L-Tartaric acid). (d) Current density at a potential of 2.03V vs RHE (overpotential of 0.8V) of only Ni-foam, achiral Ni-Au (only metal), achiral Ni-Au (Racemic-tartaric acid), and chiral Ni-Au (L-tartaric acid) coating.

Figure S7 presents SEM images of the surfaces prepared.

(A)

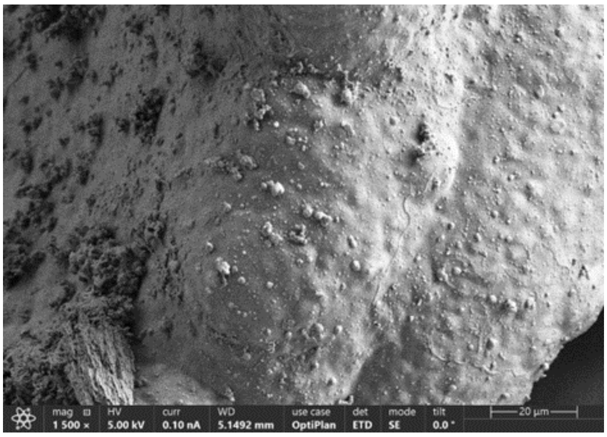

(B)

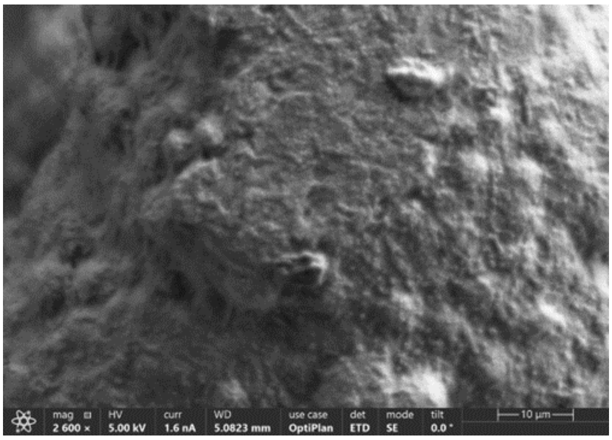

(C)

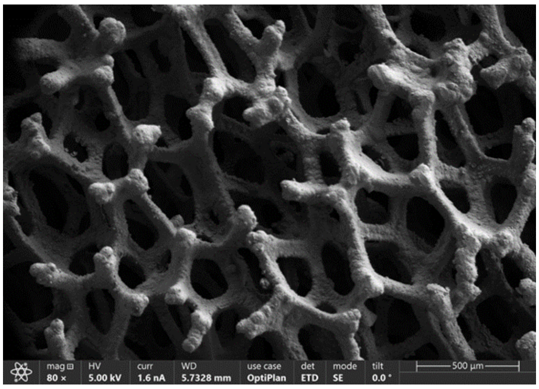

Figure S7: SEM images of the different substrates. A) Achiral Ni-Au on Ni-Foam (Only Metal). B) Chiral Ni-Au on Ni-Foam (L-Tartaric Acid), C) Zoom out of the Ni foam coated with the chiral Ni/Au.
